# *Hoya* of the Philippines part I. *Hoya migueldavidii* (Apocynaceae, Asclepiadoideae), a new species from Northern Mindanao, Philippines

**DOI:** 10.3897/phytokeys.80.12872

**Published:** 2017-06-05

**Authors:** Derek D. Cabactulan, Michele Rodda, Reynold Pimentel

**Affiliations:** 1 48 Corrales and 1st Street, Nazareth, Cagayan de Oro City, Philippines; 2 Herbarium, Singapore Botanic Gardens, National Parks Board, 1 Cluny Road, Singapore 259569; 3 Camp Phillips, Manolo Fortich, Bukidnon, Philippines

**Keywords:** Acanthostemma, Marsdenieae, waxflower

## Abstract

A new species of *Hoya* R.Br. from Mindanao (Philippines), *Hoya migueldavidii* Cabactulan, Rodda & Pimentel, is described and illustrated. It is a member of Hoya section Acanthostemma (Blume) Kloppenb. that is particularly speciose in the Philippines.

It is compared with the similar *Hoya loheri* Kloppenb, also endemic of the Philippines, from which it differs in indumentum of the vegetative parts (pubescent vs. glabrous), the shape of the corolla (almost spherical vs. partly flattened) and the type of gynostegium (not stipitate vs. stipitate)

## Introduction

*Hoya* R.Br., with an estimated 350–450 species (Rodda 2015) is the largest genus of Apocynaceae. The Philippines are, together with Borneo and New Guinea one of the centres of diversity of the genus. The number of taxon descriptions in the Philippines have been steadily increased since 2012. Kloppenburg et al. (2012) recorded more than 80 species of *Hoya* occurring in the Philippines, number that jumped to 104 species according to Aurigue et al. (2013). At present the Co’s Digital Flora of the Philippines website (Pelser et al. continuously updated, accessed on 17 March 2017) lists 121 taxa including species and subspecies. The steep increase in species number is almost entirely due to the establishment of the e-journal Hoya New, where Dale Kloppenburg started publishing new taxa since 2013 either as sole author or in collaboration with numerous Philippine botanists and growers.

In comparison to the two other centres of diversity of *Hoya*, Borneo and New Guinea, the pattern of species discovery and description in the Philippines is very different. Borneo, with 72 recorded species (Lamb and Rodda 2016) saw a peak of species descriptions in the early 2000s mostly due to the work of Dale Kloppenburg and Ted Green, and more recently a second peak in 2014 and 2015, mostly due to the work of Lamb et al. (2014) and Rodda, in preparation for the book ‘A Guide to Hoyas of Borneo’ by Lamb and Rodda (2016). New Guinea has 85 species and one subspecies (Forster 1996, Simonsson Juhonewe and Rodda 2017), mostly described in the first half of the last century, the majority of which described by Schlechter (1913) based on his own collections. Ten species and one subspecies were instead named following recent intensive fieldwork in New Guinea (Simonsson Juhonewe and Rodda 2017).

The majority of the recent publications of new Philippine *Hoya* species are based on collections from Luzon and several parts and islands in Visayas and fewer from Mindanao. The first and last authors recently made extensive collections in the island including several unidentified species. Sterile plants were also collected and brought to cultivation in the private plant nursery of last author in Bukidnon and are awaiting to bloom so that they can be studied and identified.

The new species here published is among the first cultivated collections that flowered. It belongs to Hoya section Acanthostemma, that is particularly species-rich in the Philippines and is characterised by corolla lobes revolute, outer corona lobes bilobed and pollinaria with broad, spathulate caudicles.

## Materials and methods

The description of the new species is based on the observation of the living specimens collected from the wild and cultivated at the nursery of the last author at Del Monte, Camp Phillips, Manolo Fortich, Bukidnon. Flowers were dissected and examined under the stereomicroscope and pictures were taken before pressing. Specimens of *Hoya* at A, BK, BKF, BISH, BM, BRUN, CMUH, FI, G, K, KEP, KUN, HBG, IBSC, L, M, MO, P, SAN, SAR, SNP, SING, TO, UC, US, W, WRSL and WU herbaria as well as type images at https://plants.jstor.org/ were also studied.

## Species treatment

### Hoya migueldavidii

Taxon classificationPlantaeGentianalesApocynaceae

Cabactulan, Rodda & Pimentel sp. nov.

urn:lsid:ipni.org:names:77163235-1

Fig. 1 , 2

#### Diagnosis.

Among Philippine *Hoya* species similar to *Hoya loheri* in inflorescence type (positively geotropic, convex) but separated because *Hoya loheri* has a flattened, turban-shaped corolla (vs. almost round in *Hoya migueldavidii*) and leaves and stems are entirely glabrous (vs. pubescent in *Hoya migueldavidii*)

**Figure 1.**
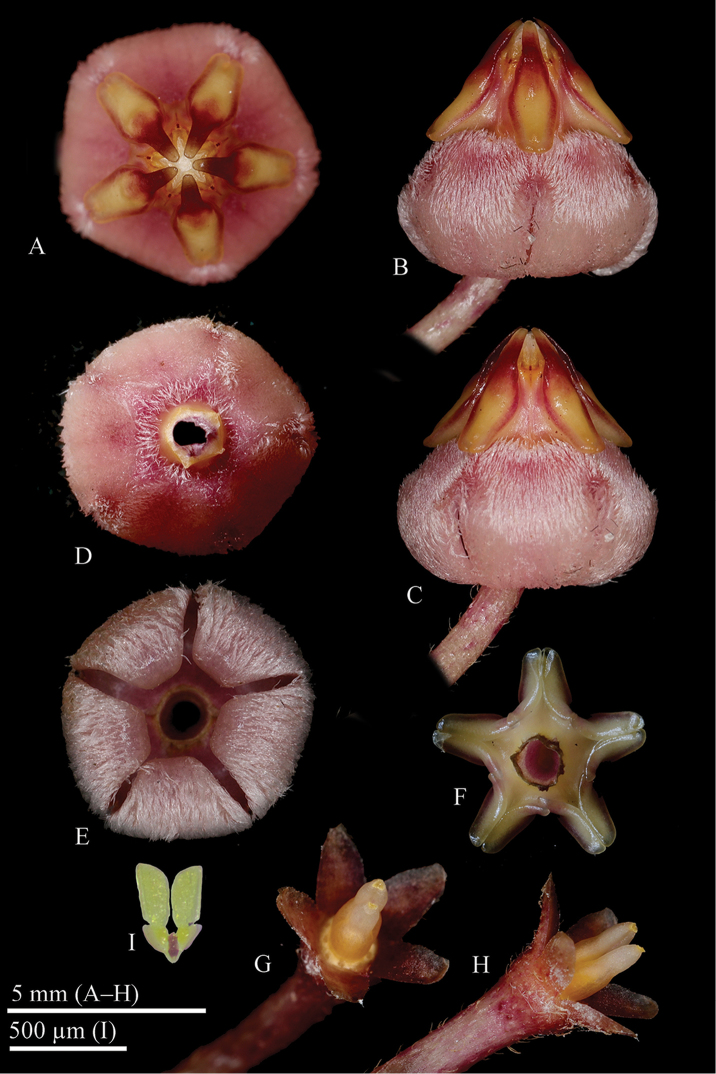
*Hoya migueldavidii* photographed from *R. Pimentel s.n.* (CMUH) prior to pressing **A** A single flower, front view **B, C** Corolla, side view **D** Corolla, with removed corona **E** Revolute margins of the corolla lobes **F** Corona, from underneath **G, H** Pedicel, calyx and ovary **I** Pollinarium (Photographs by M.D de Leon)

**Figure 2.**
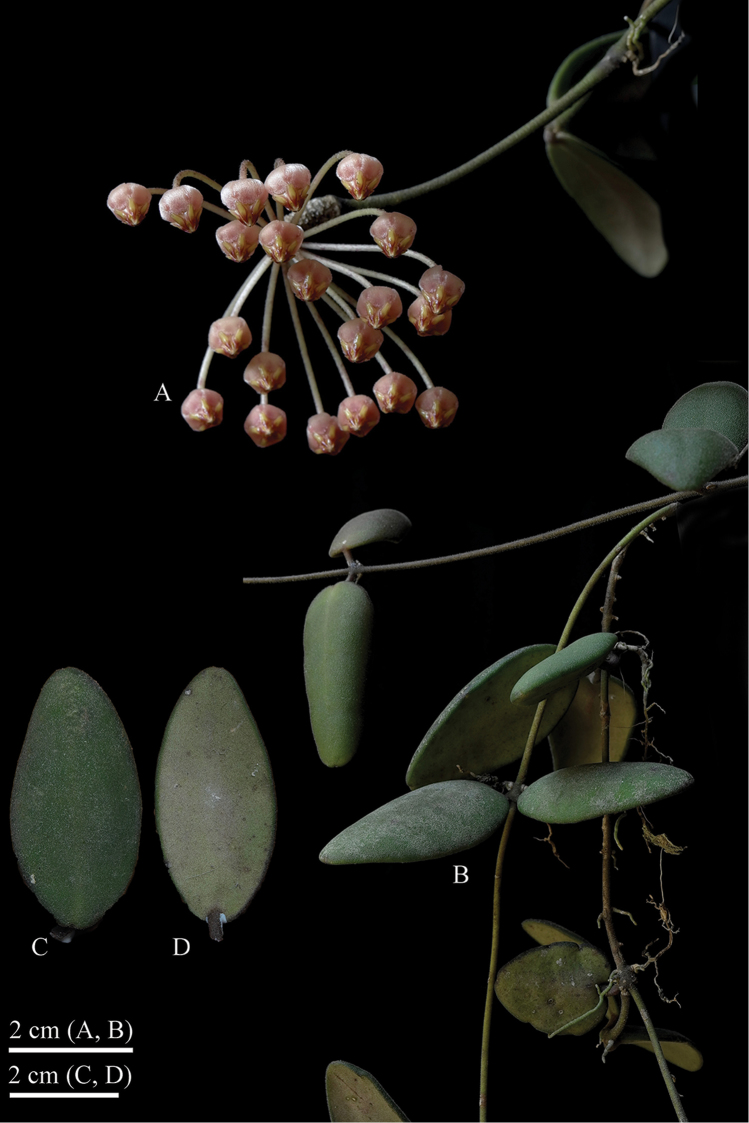
*Hoya migueldavidii* photographed from *R. Pimentel s.n.* (CMUH) prior to pressing **A** Inflorescence **B** Branch **C, D** leaf (**C** adaxial surface **D** abaxial surface). (Photographs by M.D de Leon).

#### Type.

Philippines, Mindanao, Bukidnon, Mount Kitanglad, 11 Aug 2016, *R. Pimentel s.n.* (CMUH, holotype, sheet number CMUH 827; SING, isotype).

#### Description.

Epiphytic scandent vine rooting along the stems, *roots* adventitious on internodes and just below the nodes; *stems* slender, terete, 3.0–5.0 mm diameter, green or maroon, densely strigose, *internodes* 2.5–9 cm long. *Leaf blades* fleshy, stiff, slightly concave, variable in shape, from lanceolate, elliptic-lanceolate to ovate-elliptic, 2–7.5 × 1.80–2.50 cm base rounded, apex obtuse, dark green or maroon, reddish pigmentation often occurs on the underside of the leaves, margins thickened, venation pinnate, secondary veins not visible on dry specimens, adaxially and abaxially papillate-strigose, basal *colleters* 1-3 at each lamina base, ovoid, 0.15–0.20 mm long × c. 0.15 mm in diameter, dark brown; *petiole* thickened, terete, 1–2.3 × 0.28–0.30 mm diameter, flattened approaching the lamina base, usually curved, dark green or maroon, densely strigose. *Inflorescences* extra-axillary, umbelliform, slightly concave, up to 25 flowered. *Peduncles* terete, positively geotropic, 2–8 cm long, strigose, rachis up to 3 cm long. *Pedicels*, terete, 2.50–3.80 cm × 0.70–0.72 mm in diameter, sparsely strigose, the outer pedicels strongly curved. *Calyx* lobe triangular, oblong, 0.80–1.3 mm long × 0.80–1.00 mm wide, red, outer surface strigose, inner surface glabrous. *Corolla* revolute, 5–5.3 mm diameter, 8.5–9 mm diameter when flattened, red to pink; *corolla lobes* reflexed, triangular ovate, 2.6 × 4.6 mm long, apex acute-acuminate, inside silky-pubescent, tip glabrous, outside glabrous. *Gynostegium* stipitate; *column* cylindrical, 0.04 × 0.03 mm diameter; *corona* staminal, 2.3–3.0 mm high, 4–5 mm in diameter; *lobes* ovoid-spathulate, 3.0–3.3 × 0.30–0.35 mm wide, inner processes erect above the anthers, almost linear, red, outer process long bilobed, with basal revolute margins. *Pollinia* erect, oblong, 300–320 µm long and 115–130 µm wide with a sterile edge along the outer margin; *translator arms* c. 60 µm long, each with a rounded projections c. 60 µm diam.; *corpusculum* oblong 90–110 × 35–50 µm. *Ovary* conical with an acute tip, c. 1.5 × 0.6 mm at the base, ventricose, glabrous. *Fruit* and *seed* not seen.

#### Etymology.

*Hoya migueldavidii* is named after Dr. Miguel David de Leon, viteoretina surgeon and plant and wildlife conservationist.

#### Distribution and ecology.

This new species was only once collected in Mindanao Island, Philippines but the full distribution is still unknown. It is an epiphytic climber, growing at about 1000 m in disturbed primary broad leaf forest in full sun to part shade.

#### Conservation status.

The forested area where *Hoya migueldavidii* was collected is threatened by habitat destruction due to extensive farming, charcoal production, land conversion and illegal logging. However, the species is only known from a single collection and therefore the conservation status is proposed as Data Deficient (DD, IUCN 2016) until more information is known about its area of occurrence.

#### Notes.

**Figure 3.**
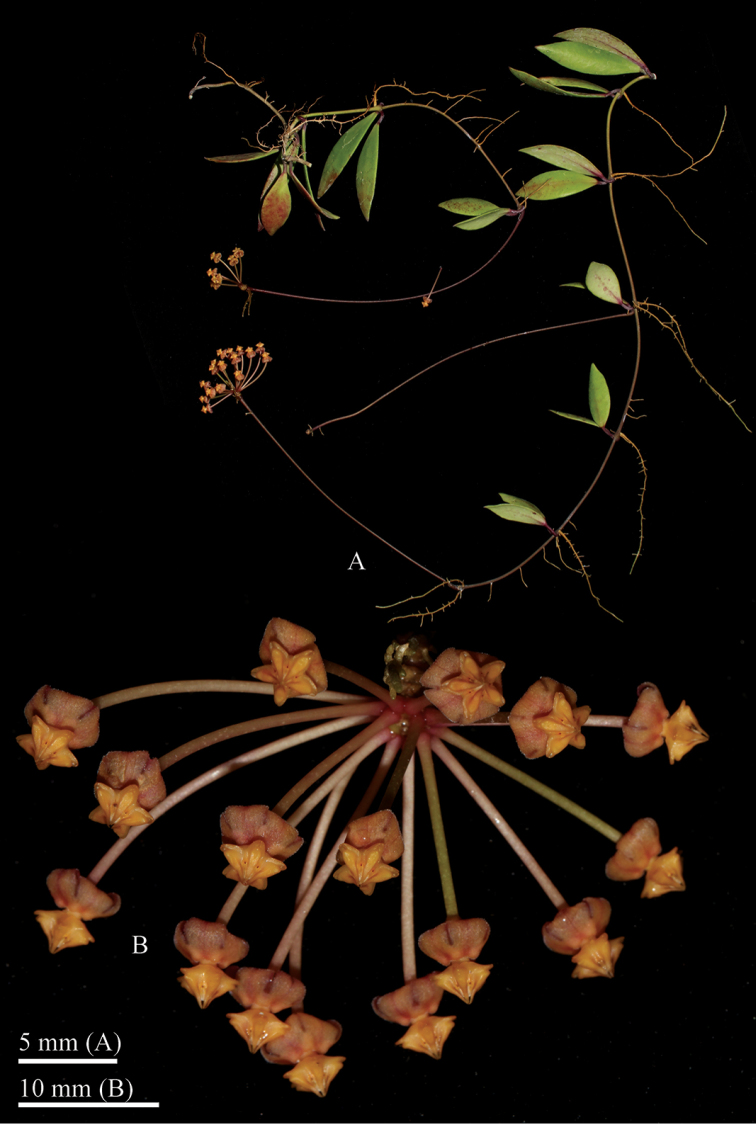
*Hoya loheri* photographed from *Rodda M MR748* (SING) prior to pressing **A** Leafy branch and inflorescence **B** Inflorescence. (Photographs by M. Rodda)

The long peduncles, shape of the inflorescences and the slender pedicels of *Hoya migueldavidii* are similar to those of *Hoya loheri* (Fig. 3). The two species can be separated because of the indumentum of the vegetative parts that are pubescent throughout in *H. migueldavidii* vs. glabrous in *H. loheri*; the shape of the corolla that is revolute and almost spherical in *H. migueldavidii* and instead revolute yet flattened in *H. loheri*; the gynostegium is sessile in *H. migueldavidii* whereas it is stipitate in *H. loheri*. Additionally the leaves of *H. migueldavidii* are similar to those of *Hoya isabelchaniae* Rodda & Simonsson from Sulawesi (Indonesia) both in shape, convex ovate-elliptic (to round in *H. isabelchaniae*) and in indumentum (pubescent). However, *H. migueldavidii* has smaller flowers of c. 7 mm vs. 8–10 mm in diameter in *H. isabelchaniae*) and different pubescence of the corolla (finely pubescent vs. setose in *H. isabelchaniae*).

#### Specimens of *Hoya loheri* examined.

*Hoya loheri*. Philippines, unknown locality, cultivated in Thailand, Chonburi, Nong Nooch Tropical Garden vouchered on 22 September 2014, *M. Rodda MR748* (SING). Luzon, Rizal Province, Paningtingan. 15 March 1915 *A. Loher s.n.* (UC [UC229373])

## Supplementary Material

XML Treatment for Hoya migueldavidii
